# Supplementary material for: Identification of photoperiod-induced specific miRNAs in the adrenal glands of Sunite sheep (Ovis aries)
Source: Front Vet Sci. 2022 Jul 22;9:888207. doi: 10.3389/fvets.2022.888207 (PMC9354845; doi:10.3389/fvets.2022.888207)
Supplement: Supplementary file 1 [file Data_Sheet_1.ZIP › Supplementary file/miRDeep2 file.zip!/miRDeep2 file/Know_Structure/LP42A3/oar-let-7i.pdf]

miRBase precursor : oar-let-7i
Total read count : 423065
oar-let-7i read count : 422891
remaining reads : 174

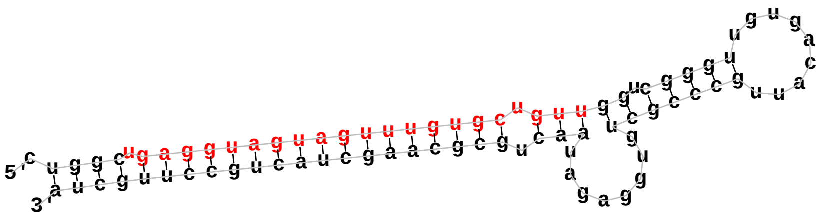

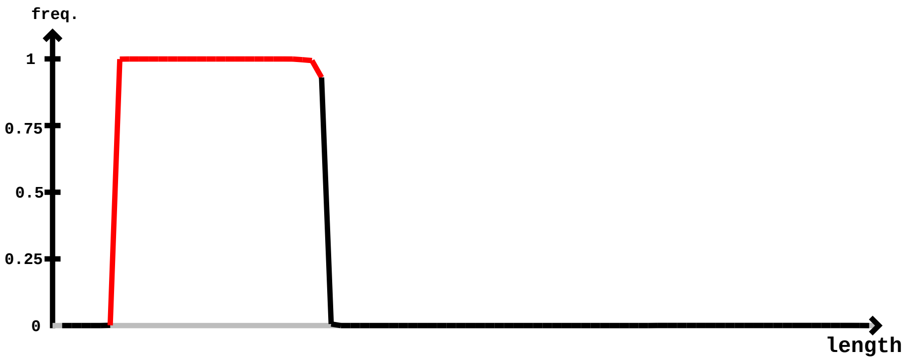

oar-let-7i

```
5'- cuggcugagguaguaguuugugcuguuggucggguugugacauugcccgcuguggagauaacugcgcaagcuacugccuugcua   -3'    exp
    .((((.(((((((((((((((((.(((((.(((((.........)))))))........))).))))))))))))))))))))    reads      mm         sample
    ...gcugagguaguaguuugugcugu..........................................................    1          0          seq
    ....cugagguaguaguuugugcu............................................................    3          0          seq
    ....cugagguaguaguuugugcug...........................................................    1          0          seq
    ....cugagguaguaguuugugcugu..........................................................    48         0          seq
    ....cugagguaguaguuugugcuguu.........................................................    381        0          seq
    ....cugagguaguaguuugugcuguug........................................................    1          0          seq
    .....ugagguaguaguuugug..............................................................    5          0          seq
    .....ugagguaguaguuugugA.............................................................    8          1          seq
    .....ugagguaguaguuugugc.............................................................    55         0          seq
    .....ugagguaguaguuugugcu............................................................    1090       0          seq
    .....ugagguaguaguuugugcug...........................................................    1310       0          seq
    .....ugagguaguaguuugugcugu..........................................................    26793      0          seq
    .....ugagguaguaguuugugcuguu.........................................................    390506     0          seq
    .....ugagguaguaguuugugcuguug........................................................    2474       0          seq
    .....ugagguaguaguuugugcuguugg.......................................................    7          0          seq
    .....ugagguaguaguuugugcuguuggu......................................................    1          0          seq
    ......gagguaguaguuugugcugu..........................................................    10         0          seq
    ......gagguaguaguuugugcuguu.........................................................    192        0          seq
    ......gagguaguaguuugugcuguug........................................................    1          0          seq
    .......agguaguaguuugugcuguu.........................................................    4          0          seq
    .............................................................cugcgcaagcuacugccuug...    4          0          seq
    .............................................................cugcgcaagcuacugccuugc..    55         0          seq
    .............................................................cugcgcaagcuacugccuugcu.    114        0          seq
    .............................................................cugcgcaagcuacugccuugcua    1          0          seq
```
